# *In vitro* transcribed sgRNA causes cell death by inducing interferon release

**DOI:** 10.1007/s13238-018-0605-9

**Published:** 2019-01-07

**Authors:** Wei Mu, Na Tang, Chen Cheng, Wen Sun, Xiaofei Wei, Haoyi Wang

**Affiliations:** 1 State Key Laboratory of Stem Cell and Reproductive Biology, Institute of Zoology, Chinese Academy of Sciences, Beijing, 100101 China; 2 University of Chinese Academy of Sciences, Beijing, 100049 China; 3 School of Life Sciences, University of Science and Technology of China, Hefei, 230026 China; 4 Beijing Cord Blood Bank, Beijing, 100176 China; 5 Institute for Stem Cell and Regeneration, Chinese Academy of Sciences, Beijing, 100101 China

**Dear editor**

The clustered regularly interspaced short palindromic repeats (CRISPR) and the CRISPR-associated proteins 9 (Cas9) systems are powerful tools for gene editing. Ribonucleoprotein (RNP) complex composed of Cas9 protein and sgRNA binds to specific genomic loci and generate DNA double strand breaks. While plasmids expressing Cas9 protein and sgRNA are routinely transfected into various cell lines to perform gene editing (Cong et al., 2013; Mali et al., 2013; Ran et al., 2013), direct delivery of Cas9-sgRNA RNP has shown higher efficiency and lower off-target effects (Kim et al., 2014), especially in human primary cells such as T cells (Hendel et al., 2015; Schumann et al., 2015). SgRNA can be generated by either *in vitro* transcription (IVT) or chemical synthesis. IVT is widely used to generate sgRNAs, since it can be easily performed in most labs.

Gene editing of human primary cells, such as human CD34^+^ hematopoietic stem and progenitor cells (HSPCs), T cells and chimeric antigen receptor T (CAR-T) cells, is important for studying gene functions in these cell types, and holds great promise to further improve cell therapy (Schumann et al., 2015; Dever et al., 2016; Liu et al., 2016; Ren et al., 2017; Zhang et al., 2017). Our previous study showed that multiplex gene editing using CRISPR-Cas9 RNP hampered the proliferation of CAR-T cells (Liu et al., 2016). It was also reported that Cas9/hCD45sg1 RNP-treated human HSPCs had lower cell number compared to Cas9 protein-treated control (Gundry et al., 2016). In this study, we investigated the mechanism of cell loss after electroporation of Cas9-sgRNA RNP and find an easy method to resolve it.

As our initial attempt of gene editing in human CD34^+^ HSPCs, we delivered Cas9-sgRNA RNPs into HSPCs by electroporation, using sgRNA *in vitro* transcribed by T7 polymerase. We observed significantly decreased cell number and reduced CD34 expression in survived cells 48 h after electroporation in experiments using five different sgRNA (Fig. S1A and S1B). Accordingly, the colony forming ability of HSPCs after RNP electroporation was markedly compromised (Fig. S1C). To identify the factor that contributed to this effect, we delivered IVT sgRNA (sgRNA-IVT), RNP complex consisting of Cas9 protein and IVT sgRNA (RNP-IVT), or Cas9 protein alone individually into primary HSPCs by electroporation. We observed lower cell viability, reduced CD34 expression and decreased colony forming ability in sgRNA-IVT and RNP-IVT groups, while the cells in Cas9 protein group and mock electroporation group survived well (Fig. 1A–C), indicating that sgRNA-IVT reduced HSPC stemness in addition to causing cell death.

**Figure 1:**
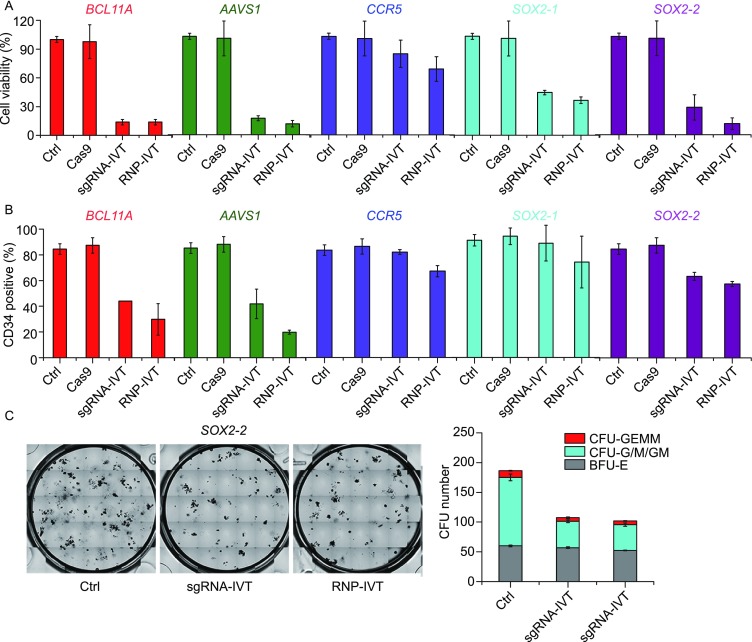

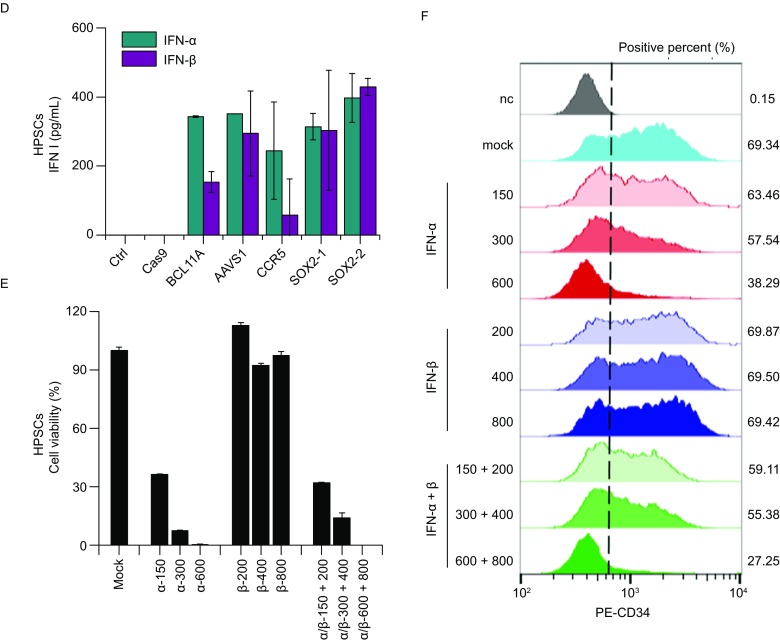
**IVT sgRNA induced the production of IFN-α, which lead to severe cell death and stemness reduction in human CD34**^**+**^ **HSPCs**. (A–C) Cell viability (A), CD34 expression (B) and colony forming units (C) of human primary HSPCs electroporated with indicated samples. Cell viabilities and CD34 expression were measured 48 h post electroporation by cell counting and FACS respectively. For colony forming unit (CFU) assay, same number of viable cells were seeded 48 h post electroporation, the colony number and lineage differentiation were counted and analyzed two weeks later. In (A), (B), (C) experiments were performed in HSPCs from two donors. Error bars represent SD for two biological replicates. (D) Type I IFN production in HSPCs after electroporation. RNPs targeting indicated sites were delivered into HSPCs. The IFN concentration in medium supernatant were measured 24 h post electroporation by ELISA, respectively (mean ± SD, *n* = 3). (E and F) The cell viability (mean ± SD, *n* = 3) (E) and CD34 expression (F) of HSPCs after co-culture with IFN in different concentrations

To validate whether sgRNA could also cause the death of T cells, we electroporated different amounts of IVT sgRNA into human primary CD3^+^ T cells. Reduced cell viability and increased immune stimulation were observed when higher amounts of sgRNAs were used (Fig. S2A).

All sgRNAs we tested had a negative effect on the survival of HSPCs and T cells, suggesting that some common feature of these IVT sgRNA was responsible for this effect. Based on existing reports, we hypothesized that the 5’ triphosphate of IVT sgRNA induced type I IFN production, leading to cell death in HSPCs and T cells. To test this hypothesis, we electroporated sgRNA-IVT targeting different genomic loci into CD34^+^ HSPCs and CD3^+^ T cells respectively, and measured the concentration of type I IFN in the culture medium using enzyme-linked immunosorbent assay (ELISA). Indeed, we detected significant release of IFN I in all samples electroporated with RNP-IVT (Figs. 1D and S2B).

Next we explored the effects of type I IFN on human primary cells, we cultured freshly isolated HSPCs or CD3^+^ T cells with IFN I at different concentration. We found that the survival and CD34 expression of HSPC were decreased drastically along with the increased concentration of IFN-α. However, IFN-β had no obvious effect (Fig. 1E and 1F). Similar results were obtained in CD3^+^ T cells (Fig. S3A). Using Caspase-Glo^R^ 3/7 assay, we detected significantly increased caspase activity in T cells cultured with IFN-α (Fig. S3B). These data confirmed that IVT sgRNA induced IFN-α release, which led to the apoptosis of primary cells.

To verify whether the 5’ triphosphate of sgRNA induced the production of IFN I, we removed the 5’ triphosphates of sgRNA using calf intestine phosphatase (CIP) (Dong-Ho et al., 2004) and chemically synthesized a 5’-hydroxylated (5’-OH) sgRNA targeting *AAVS1* site. The viability of HSPCs and T cells electroporated with CIP-treated and chemically synthesized sgRNA together with Cas9 protein were similar to control, which was significantly higher than RNP-IVT samples (Fig. S4A and S4B). Consistently, the caspase activity induced by sgRNA without 5’ triphosphate was much lower compared with sgRNA containing 5’ triphosphate (Fig. S4C and S4D). These results demonstrated that the 5’ triphosphate of sgRNA is the major factor that induced innate immunity and primary cell death.

To confirm this finding at multiple genomic loci, we electroporated IVT sgRNA with CIP treatment alone or in RNP complex, targeting *BCL11A* and other four sites, into HSPCs. Indeed, CIP-treated sgRNA (sgRNA-CIP) did not induce IFN release (Figs. 2A and S5A), and led to significantly improved survival rate and colony formation capability of HSPCs, to a level comparable to the mock electroporation control (Figs. 2B, 2C, S5B and S5C). To detect the stemness of electroporated HSPCs more comprehensively, we further analyzed the colony formation capability of granulocyte, erythroid, macrophage and megakaryocyte (GEMM) lineage and the *in vivo* chimeric ability. We could see the numbers of CFU-GEMM and the chimeric efficiency both improved significantly in CIP treated group contrast to IVT sgRNA group (Figs. 2D, 2E and S6). From above data, we suggested that CIP treatment of IVT sgRNA not only increased cell survival, but also maintained the stemness of HSPCs.

**Figure 2:**
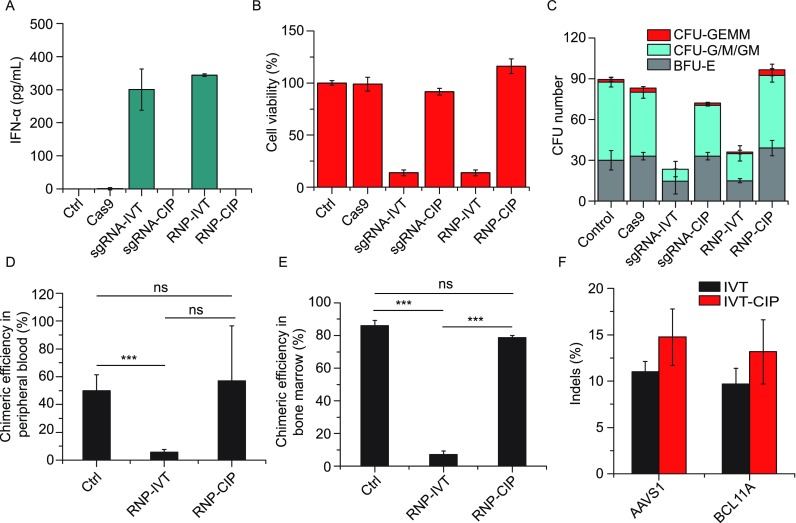
**Removing 5’ triphosphate of IVT sgRNA by CIP completely rescued the detrimental effects in HSPCs**. (A) CIP treatment reduced the production of IFN-α production in HSPCs. (B and C) The cell viability (B) and colony formation ability (C) of HSPCs with CIP treatment were comparable with electroporation mock control. Experiments were performed in cells from two donors, error bars represent SDs for two biological replicates. (D and E) The chimeric efficiencies of HSPCs in NPG mice peripheral blood (D) and bone marrow (E). The chimeric efficiencies were collected 12 weeks post injection. Error bars represent SD for two biological replicates. (F) CIP treatment did not affect the gene editing efficiency of CRISPR-Cas9 system in HSPCs. Indel frequencies were measured by TIDE and bars represent average indel frequencies ±SD, *n* = 3. Ctrl: control; RNP-CIP: CIP treated *BCL11A* IVT sgRNA + Cas9 protein; RNP-IVT: *BCL11A* IVT sgRNA + Cas9 protein. *P* values were calculated by employing unpaired *t*-test comparing with indicated groups. *** *P* < 0.001

Similar to HSPCs, CIP treatment of IVT RNP (RNP-CIP) targeting various genomic loci also attenuated immune-stimulation and improved cell viability of CD3^+^ T cells and anti-CD19 CAR-T cells (Figs. S7 and S8). These results demonstrated that the 5’ triphosphate of IVT sgRNA induced type I IFN production, which led to cell death; removing the 5’ triphosphate using CIP could rescue these detrimental effects.

We also explored the underlying mechanism of IVT sgRNA induced IFN I release using HeLa cells. We observed the phosphorylation of IRF3 and the expression levels of *IFNB1*, *IFIT1*, *MDA5* and *RIG-I* were significantly up-regulated in sgRNA-IVT and RNP-IVT electroporation groups (Figs. S9A, S9B, S9C and S10), confirming the innate immunity was also activated in HeLa cells.

Next, we established *MDA5* and *RIG-I* knockout HeLa cell lines (Fig. S9D). Compared with wild type (WT), the expression of *IFNB1* and *IFIT1* in *MDA5* KO was markedly decreased, and was comparable to mock, *RIG-I* KO and *MDA5*/*RIG-I* DKO clones after IVT sgRNA electroporation (Fig. S9E). These results suggested that *MDA5* and *RIG-I* recognized the IVT sgRNA and induced IFN expression.

We next assessed the effect of removing 5’ triphosphate of IVT sgRNA via CIP on gene editing efficiency. Firstly, we found that the removal of 5’ triphosphate did not significantly affect the persistence of sgRNA in 293T cells (Fig. S11A). We further analyzed the gene editing efficiency of sgRNA-CIP, and found that CIP treatment did not affect the gene editing efficiency of the CRISPR-Cas9 system in HSPCs, T cells, CAR-T cells and various cell lines (Figs. S11B, S11C and S12). Furthermore, performing multiplex gene editing in anti-CD19 CAR-T cells using sgRNA -CIP did not affect the function of CAR-T cells (Fig. S13).

In this study, we showed that the triphosphate at 5’ end of IVT sgRNA triggered the innate immunity through *RIG-I*/*MDA5* signaling pathway and induced the production of type I IFN, which led to the death of CD34^+^ HSPCs and CD3^+^ T cells, and stemness reduction in CD34^+^ HSPCs. By treating the IVT sgRNA with CIP, we were able to eliminate the IFN induction effect of IVT sgRNA, and achieved significantly better cell survival without affecting gene editing efficiency of the CRISPR-Cas9 system. While our manuscript is under review, another group published similar findings in HeLa and T cells, and identified RIG-I as the sensor for IVT sgRNA (Kim et al., 2018). In addition to T and CAR-T cells, our work demonstrated a dramatic effect on both the survival and stemness of human HSPCs, and identified MDA5 as another player involved in innate immunity response induced by IVT sgRNAs.

Although sgRNA generated by chemical synthesis is an option, the generally high costs and long production time limit their widespread application. Moreover, the length of sgRNA is almost reaching the current limits of RNA chemical synthesis. With various optimization and added structures and sequences, sgRNAs longer than 100 nt are commonly used (Shechner et al., 2015; Cheng et al., 2016) and are most conveniently generated by IVT. Therefore, RNA-CIP is useful for a variety of applications in gene editing and transcription regulation of primary cells.

## Electronic supplementary material

Below is the link to the electronic supplementary material.

Supplementary material 1 (PDF 1928 kb)
