# Supplementary material for: In vitro transcribed sgRNA causes cell death by inducing interferon release
Source: Protein Cell. 2019 Jan 7;10(6):461–5. doi: 10.1007/s13238-018-0605-9 (PMC6538590; doi:10.1007/s13238-018-0605-9)
Supplement: Supplementary file 1 — Supplementary material 1 (PDF 1928 kb) [file 13238_2018_605_MOESM1_ESM.pdf]

Supplementary data

In Vitro Transcribed SgRNA Cause Cell Death by Inducing Interferon Release

Wei Mu[1,2※], Na Tang[1,2※], Chen Cheng[1,3※], Wen Sun[1], Xiaofei Wei[4], Haoyi Wang[1,2*]

Running title: IVT SgRNA reduced cell viability and stemness of HSPCs

Keywords: CRISPR-Cas9, Cell death, IFN, Innate immunity, CIP

*To whom correspondence should be addressed:

Haoyi Wang, Ph.D.

State Key Laboratory of Stem Cell and Reproductive Biology

Institute of Zoology, Chinese Academy of Sciences

Email: wanghaoyi@ioz.ac.cn

[1]State Key Laboratory of Stem Cell and Reproductive Biology, Institute of Zoology, Chinese Academy of Sciences, Beijing, 100101, China

[2]University of Chinese Academy of Sciences, Beijing, 100049, China

[3]School of Life Sciences, University of Science and Technology of China, Hefei, Anhui, 230026， China

[4]Beijing Cord Blood Bank, Beijing, 100176, China

MATERIALS AND METHODS

Cell Lines. Human 293T and Hela cells were maintained in DMEM (Gibco) supplemented with 10% (v/v) FBS, 100 U/mL penicillin and streptomycin. The CD19 and luciferase expressing K562 and Jukat cells were maintained in RPMI1640 medium (Gibco) supplemented with 10% (v/v) FBS, 100 U/mL penicillin and streptomycin. All cell lines were cultured at 37 ℃ in a 5% CO_2 atmosphere.

Isolation of $CD3^+$ T cells and $CD34^+$ HSPCs from umbilical cord blood (UCB) units. Fresh UCB units were obtained from healthy volunteer donors who have provided informed consent from the Beijing Cord Blood Bank (Beijing, China), and mononuclear cells were separated with human mononuclear cells separation medium 1.007 (Beijing DongFangHuaHui Biomedical technology co., Ltd). T cells were isolated using the EasySep human T cell enrichment kit (Stemcell Technologies), activated and expanded with anti-CD3/anti-CD28 Dynabeads (Thermo Fisher Scientific) at the ratio of 1:1 according to the manufacturer's instructions. T cells were cultured in X-vivo15 medium (Lonza) supplemented with 5% (v/v) heat-inactivated fetal bovine serum (Gibco) and 300 IU/mL recombinant human IL-2 (Sino Biological Inc.). HSPCs were isolated using the human cord blood CD34 positive selection kit II (Stemcell Technologies), and cultured in stem span H3000 with expansion supplement (Stemcell Technologies). All cells were cultured at 37 ℃ in a 5% CO_2 atmosphere.

Generation of CAR-T cells. Anti-CD19 CAR-T cells were generated and expanded as previously described(Liu et al., 2016) with minor modification. Briefly, freshly purified primary $CD3^+$ T cells were activated for 24 h and then infected with lentiviruses harboring the anti-CD19 CAR. Lentiviruses were produced by co-transfecting lentiviral vector with packaging plasmids pMD2.G, psPAX2 into 293T cell and the virus supernatant was harvested 48h post transfection.

***In vitro* transcription**.

The plasmid PX330 (#42230) containing sgRNA backbone was used as PCR template and the acquired PCR amplicon containing T7 promoter, 20bp target sequence and sgRNA backbone was used as IVT template. The *in vitro* transcription was performed using MEGAshortscript T7 kit (Thermo Fisher Scientific). For the CIP (NEB) treatment, 2U enzyme was added to each μg of *in vitro* transcribed sgRNA, and further incubated at 37 °C for one hour. SgRNA were then purified with MEGAclear columns (Thermo Fisher Scientific) and eluted with elution buffer.

Electroporation of Human Primary Cells. Cas9 and sgRNA ribonucleoprotein (RNP) were prepared immediately before electroporation by incubating 6 μg Cas9 protein (provided by Shenzhen Fapon Biological Therapy Co., Ltd) with 6 μg indicated sgRNA at room temperature for 20 min. 1×10^5 cells were centrifuged at 200 g for 5 minutes and resuspended in 20 μl transfection buffer containing indicated RNP or sgRNA alone and then transferred into the electroporation cuvette. All electroporation experiments were performed using 4D-Nucleofector System N (Lonza)

and the P3 Primary Cell 4D-Nucleofector X Kit (V4XP-3024, Lonza), program EO-115 and program EO-100 was selected for $CD3^+$ T or $CD34^+$ HSPCs respectively. After electroporation, cells were resuspended in 200 μl pre-warmed medium and transferred into a 96-well cell plate and incubated at 37 ℃ in an atmosphere of 5% CO_2.

Real-time PCR. $CD3^+$ T cells, 293T cells and Hela cells were transfected with indicated sgRNA. Total RNA was extracted using the Trizol reagent (Life Technologies) 24 h post transfection. cDNA was synthesized by reverse transcription using the TransScript-Uni One-Step gDNA Removal and cDNA Synthesis Supermix Kit (TransGen Biotech). mRNA quantification of *MDA5*, *RIG-I*, *IFNB1*and *IFIT1*was performed by CFX96 real-time detection system (Bio-Rad). Housekeeping gene *GAPDH* was used as an internal control. The qPCR primers used for the amplification of target gene are listed in Supplementary Table 1.

Flow cytometry. CytoFLEX (Beckman Coulter Inc) was used to perform fluorescent expression analysis. Cells were harvested 48 h after electroporation and prepared according to the manufacturer's protocol. The antibodies used are as follows: TCR α/β-APC (IP26, Biolegend), β2-microglobulin (B2M)-APC (2M2, Biolegend), CD34-PE (BD Pharmingen).

Analysis of gene editing efficiency. The genomic disruption level of *TRAC*, *B2M*, *PD-1*, *AAVS1*, *BCL11A* and *SOX2* in T cells or *BCL11A*, *AAVS1*, *CCR5*, *SOX2* in HSPCs or *AAVS1* in 293T, Hela and Jukat T cells were determined by surveyor

nuclease assay using surveyor mutation detection kit (Integrated DNA Technologies, Inc). The percentage of target disruption was quantified by densitometry and calculated as described (Guschin et al., 2010). The indels frequency of *TRAC* and *B2M* were measured by TIDE (Tracking Indels by Decomposition) analysis(Brinkman et al., 2014) in CAR-T cells. The PCR primers used for the amplification of target loci are listed in Supplementary Table 1.

Cytokine enzyme-linked immunosorbent assay (ELISA). The amount of interferon α and β secreted into the growth medium was determined using IFN-α (Biolegend) and IFN-β (PBL) ELISA kits. The medium from indicated cells was collected 24 h after the electroporation and was assayed according to the manufacturer's protocols. Supernatants of effector cells were harvested after co-incubation with target tumor cells (CD19-luciferase expressing K562 cells) at a 1:1 ratio (4×10^4 cells each) for 16 h. Cytokines (IFN-γ， IL-2) production by effector (CAR T, KO CAR T, T) cells were evaluated by ELISA (Biolegend) according to the manufacturer's protocols.

Luciferase-based cytolysis assay. K562-CD19-luciferase cell based cytotoxicity was performed by a modified version of a luciferase-based CTL assay(Moon et al., 2014). Briefly, K562-CD19-luciferase cells and effector cells were suspended at a density of 4×10^5 cells/ml in RPMI1640 medium, then seeded in white opaque plate at the ratio of 1:1 and incubated at 37 ℃ in 5% CO_2 for 16 h. 10 μl of Steady-Glo luciferase substrate (Promega) was added, 5 min later, luminescence was recorded by PerkinElmer VICTOR X3. The results were reported as percentage of killing based on

the luciferase activity in the wells compared with tumor cells alone (% killing = 100 – ((RLU from well with effector and target cell coculture) / (RLU from well with target cells) × 100)).

Colony Forming Unit Assay. 1000 viable HSPCs were suspended immediately or 48 h post electroporation in 300 μl IMEM (Gibco) supplemented with 2% FBS, and then added the cell mix into 3 ml H4434 Methoculture™ medium (Stemcell Technology). Cells were mixed and seeded into two wells of a 6-well Smartdish (Stemcell Technology), which were then cultured at 37 ℃ with saturated humidity and an atmosphere of 5% CO_2. Colonies data were collected and analyzed by STEMvision (Stemcell Technology) two weeks later.

In vivo chimeric assay. 5-6 weeks old NOD-Prkdcscid Il2rgnull (NPG) mice (VITALSTAR, Beijing, China) were injected with 1×10^5 electroporated HPSCs via intravenous injection after 1.6 Gy X-ray irradiation. Peripheral blood and bone marrow were collected 12 weeks and 16 weeks after transplantation. Mononuclear cells were stained with anti-mouse CD45 APC and anti- human CD45-PE antibodies (Biolegend) after red blood cell lysis. The chimeric efficiency were calculated with the following formula: chimeric % = human CD45 positive %/ (human CD45 positive % + mouse CD45 positive %).

Immunoblot analysis. Hela cells were electroporated with 15 μg IVT sgRNA or CIP treated IVT sgRNA per 6×10^5 cells using SE cell line 4D-nucleofector kit, program CN-114. Cells were harvested 24 hours post transfection or electroporation and lysed

in ice-cold RIPA buffer (Beyotime, P0013B). Protein samples were separated on 12% SDS-PAGE gels and transferred to nitrocellulose membranes (Millipore). The membranes were blocked in 5% BSA in TBST (Cell Signaling Technology) and incubated with primary antibodies: anti-IRF3 (Cell Signaling Technology), anti-phospho-IRF3 (Cell Signaling Technology) and anti-GAPDH (Cell Signaling Technology) at 4 ℃ overnight. We used horseradish peroxidase linked goat anti-mouse or rabbit secondary antibodies. The immunoreactive products were detected with enhanced chemiluminescence reagent (ThermoFisher).

Caspase-Glo^{R} 3/7 Assay. Caspase 3/7 activity was determined using Caspase-Glo^{R} 3/7 assay (Promega) with modified protocol. Briefly, we used 10,000 cells per well in a 96-well plate with 100 μl medium. 12 hours after co-culture of T cells with IFN-α (100pg/ml) or 24 hours post electroporation of HSPCs and T cells,96-well plates containing cells were removed from the incubator and equilibrated to room temperature.100 μl Caspase-Glo^{R} 3/7 reagent was added into each well. The plates were incubated at room temperature for 30 minutes after gentle mix. The luminescence of each sample was recorded by PerkinElmer VICTOR X3.

Establishment of gene knockout cell lines. sgRNAs targeting *RIG-I* and *MDA5* were constructed into PX330 plasmid respectively. 1×10^{6} of Hela cells were transfected with 2 μg plasmid, 48 hours post transfection, GFP positive cells were sorted by flow cytometry. Single cell were plated into 96-well plate by limiting dilution. Genotype of

each single cell clone was measured by sequencing (TSINGKE Biological Technology) and analyzed by TIDE two weeks later.

Statistical analysis. The statistical analysis represented mean ±s.d. from three independent assays. All statistical comparisons were evaluated by unpaired one way ANOVA with *P*-value considered as ***$P < 0.001$; **$P < 0.01$; *$P < 0.05$.

Supplemental Figure Legends

Figure S1

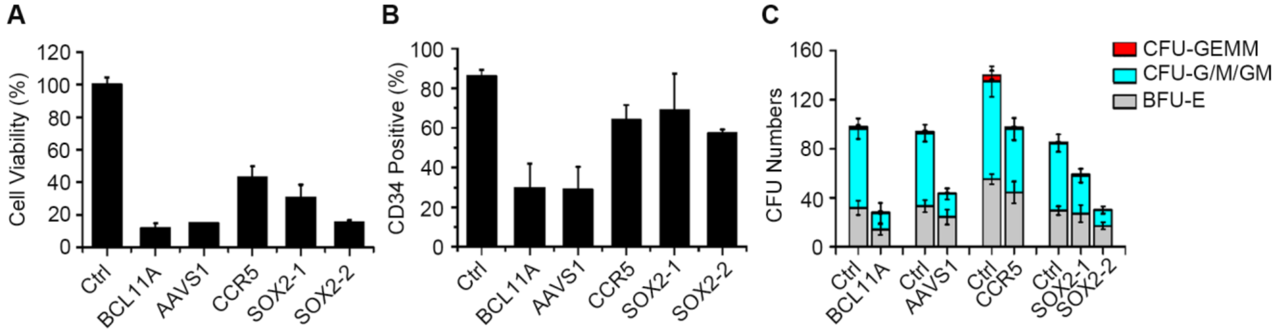

Supplemental Figure 1 | Cas9-sgRNA (RNPs) caused severe cell death and stemness reduction in human CD34$^+$ HSPCs. Cell viability **(A)** CD34 expression **(B)** and colony forming unit **(C)** of human CD34$^+$ HSPCs electroporated with indicated RNPs. Cell numbers and CD34 expression were measured 48h post electroporation by cell counting and FACS, respectively. For Colony Forming Unit (CFU) assay, same numbers of viable cells were seeded immediately post electroporation, the number and lineage differentiation of colonies were counted and analyzed two weeks later. Experiments were performed using cells from two donors. Error bars represent SD for two biological replicates.

Figure S2

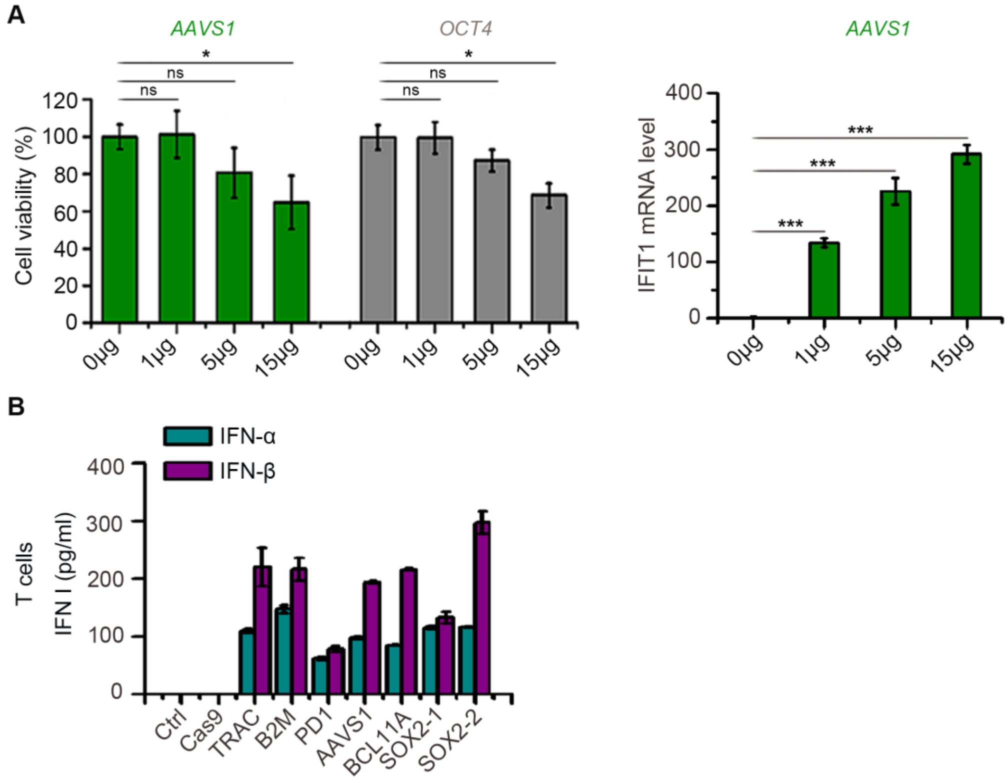

Supplemental Figure 2 | IVT sgRNA caused cell death and type I IFN release in T cells. **(A)** Cell viability (left) and immunostimulation (right) of $CD3^+$ T cells after electroporation with different amounts of IVT sgRNAs targeting *AAVS1* locus and *OCT4* promoter regions. 1×10^6 T cells were electroporated with 0, 1, 5 and 15μg IVT sgRNA respectively, cell numbers were analyzed 48h post electroporation by cell counting after stained with Trypan Blue (Mean ± SD, n = 3). The level of immune activation was represented by IFIT1 mRNA expression. **(B)** Type I IFN production in $CD3^+$ T cells after electroporation with Cas9 protein or RNPs targeting indicated loci. *P* values were calculated by employing one way ANOVA comparing the values from indicated group. $***P < 0.001$.

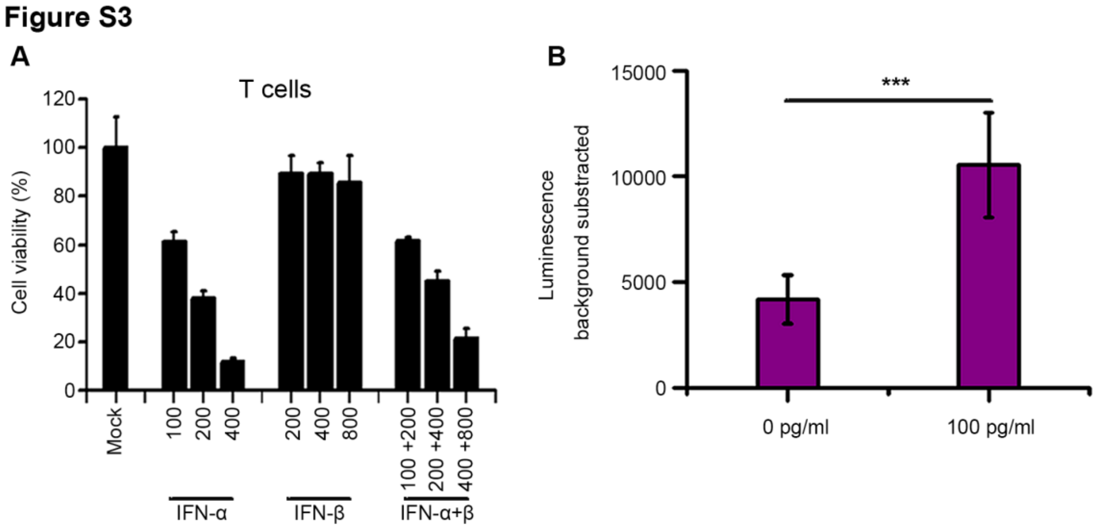

Supplemental Figure 3 | IFN-α induced severe cell death and Caspase activity increase in T cells.

(A) T The cell viability of $CD3^+$ T cells co-cultured with IFN I in different concentrations (Mean ± SD, n = 3). **(B)** The caspase activity of $CD3^+$ T cells, co-cultured with IFN-α, was detected by Caspase-Glo^R 3/7 Assay. The caspase activity of $CD3^+$ T cells increased more than 2 times 12h after adding 100 pg/ml IFN-α in the culture medium. *P* values were calculated by employing one way ANOVA comparing the values from indicated group. ***$P < 0.001$.

Figure S4

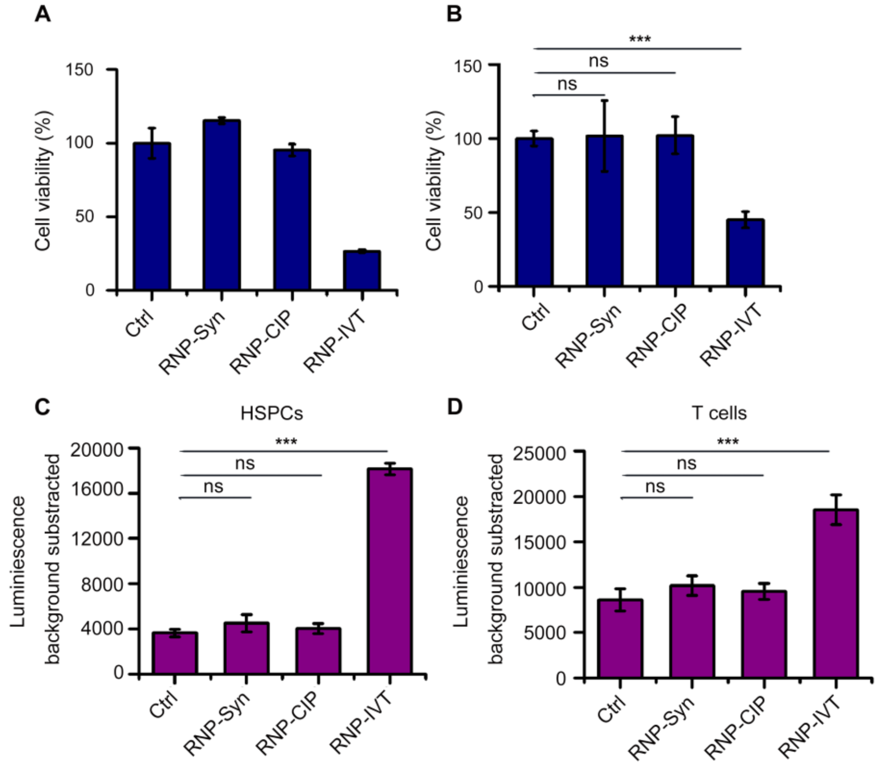

Supplemental Figure 4 | Removing 5' triphosphate of sgRNA rescued detrimental effects on cell viability.

(A, B) The cell viability was decreased significantly in *AAVS1* RNP-IVT group vs Ctrl, RNP-Syn and RNP-CIP groups in $CD34^+$ HSPCs **(A)** and $CD3^+$ T cells **(B)**. **(C)** The caspase activity of $CD34^+$ HSPCs increased more than 4 times 24h after *AAVS1* RNP-IVT electroporation. **(D)** The caspase activity of $CD3^+$ T cells increased nearly 3 times 24h after RNP-IVT electroporation. Ctrl: Control; RNP-IVT: IVT sgRNA + Cas9 protein; RNP-Syn: Chemically synthetic 5'-OH *AAVS1* sgRNA + Cas9 protein; RNP-CIP: calf intestine phosphatase (CIP) treated *AAVS1* IVT sgRNA + Cas9 protein. (Mean ± SD, n = 3). *P* values were calculated by employing an unpaired one way ANOVA comparing the values from indicated group. $^{**}P < 0.01$; $^{***}P < 0.001$; ns: no significant.

Figure S5

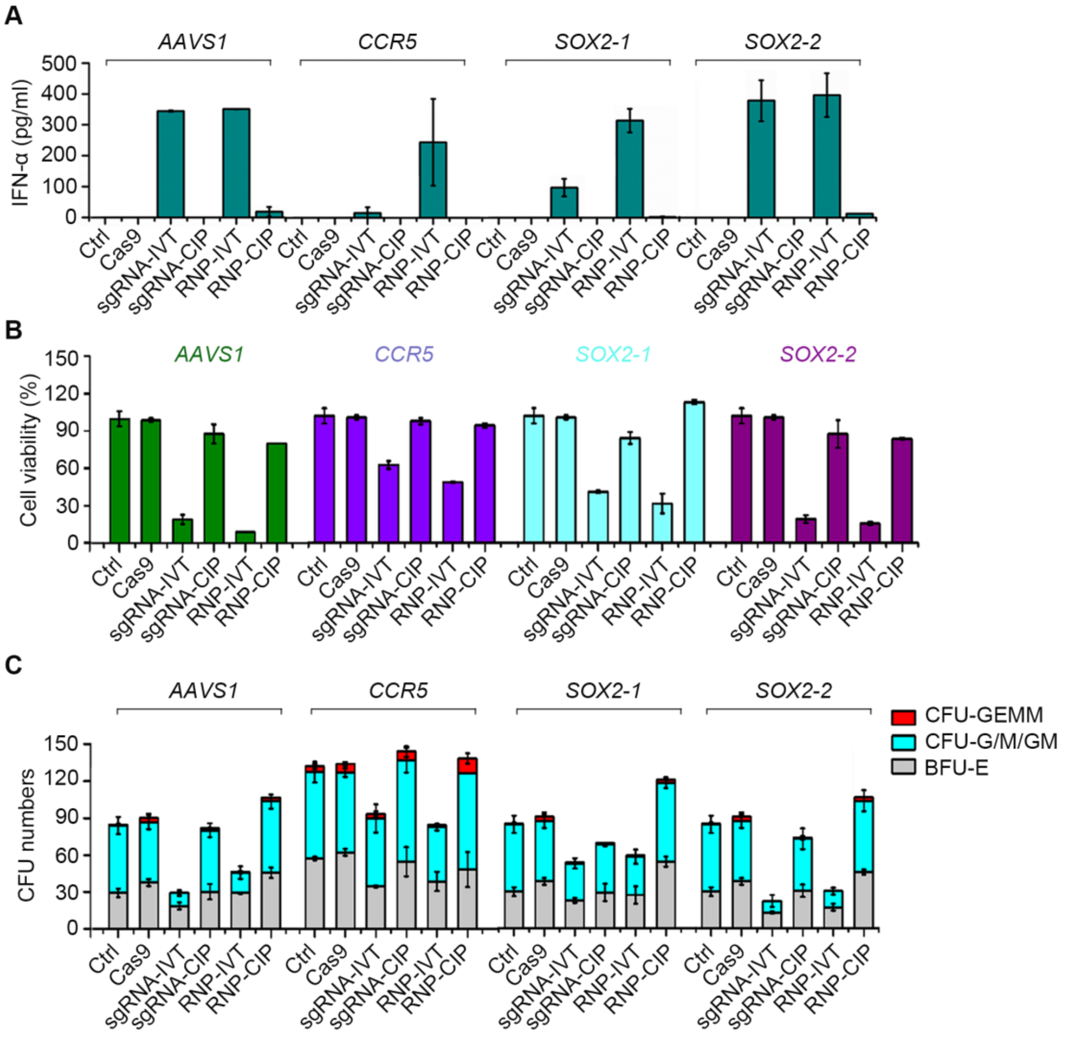

Supplemental Figure 5 | Removing 5' triphosphate of IVT sgRNA by CIP completely rescued the detrimental effects. (A) CIP treatment reduced the production of type I production in HSPCs. **(B, C)** The cell viability **(B)** and colony formation ability **(C)** of HSPCs with CIP treatment were comparable with electroporation mock control. Experiments were performed in cells from two donors, error bars represent SDs for two biological replicates. 1×10^5 cells were electroporated with 6 μg indicated sgRNAs or RNPs. The IFN concentration in medium supernatant was measured 24h, the cell viability was detected 48h and the CFU assay was conducted immediately post electroporation and collected 2 weeks later.

Fugure S6

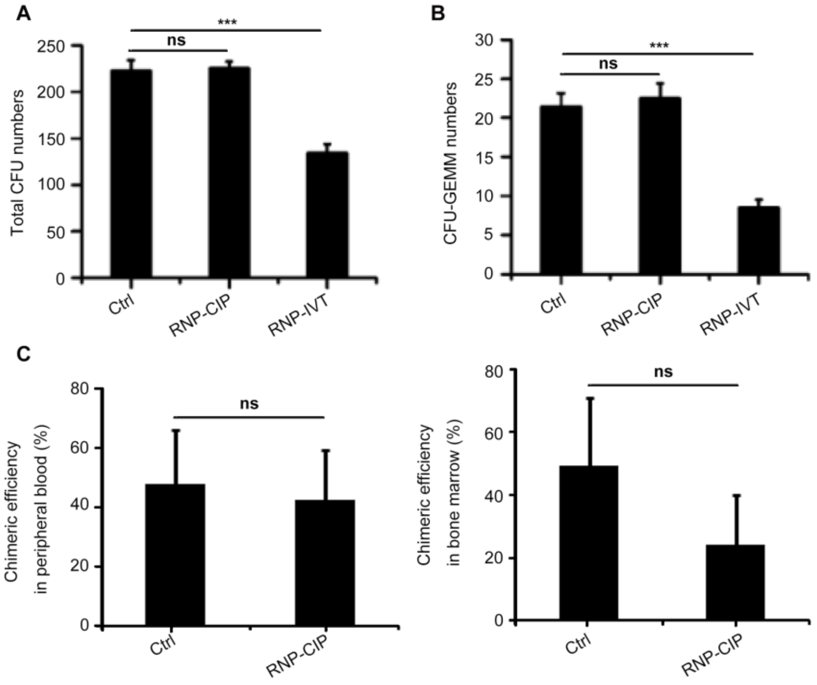

Supplemental Figure 6 | Removing 5' triphosphate of IVT sgRNA by CIP rescued detrimental effects on stemness of CD34^{+} HSPCs. Same numbers of live CD34^{+} HSPCs electroporated with indicated RNPs were plated in MethoCult^{TM} *in vitro* or injected into irradiated NPG mice *in vivo* respectively. The total CFU numbers **(A)** and CFU-GEMM numbers **(B)** decreased significantly in *BCL11A* RNP-IVT electroporation group. Error bars represent SD for 12 technical replicates. (Mean ± SD, n = 12). **(C)** The chimeric efficiencies in both peripheral blood (left pannel) and bone marrow (right pannel) were no difference between Ctrl and RNP-CIP groups. The chimeric efficiencies were analyzed 16 weeks post injection. Ctrl: control; RNP-CIP: CIP treated *BCL11A* IVT sgRNA + Cas9 protein; RNP-IVT: *BCL11A* IVT sgRNA + Cas9 protein. *P* values were calculated by employing an unpaired one way ANOVA and *t-test* comparing the values from indicated group. ***$P < 0.001$; ns: no significant.

Figure S7

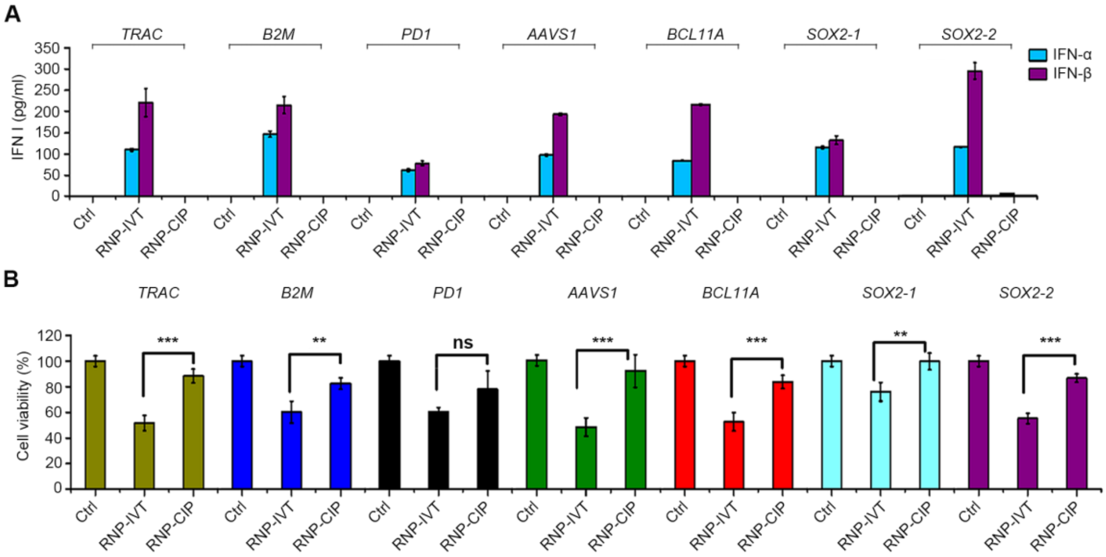

Supplemental Figure 7 | Removing 5' triphosphate of IVT sgRNA by CIP rescued detrimental effects in CD3⁺ T cells. IFN I production **(A)** and cell viability **(B)** of $CD3^+$ T cells after delivered with indicated RNPs (Mean ± SD, n = 3). *P < 0.05, **P < 0.01, ***P < 0.001; P values were calculated by employing an unpaired one way ANOVA comparing the values from indicated group.

Figure S8

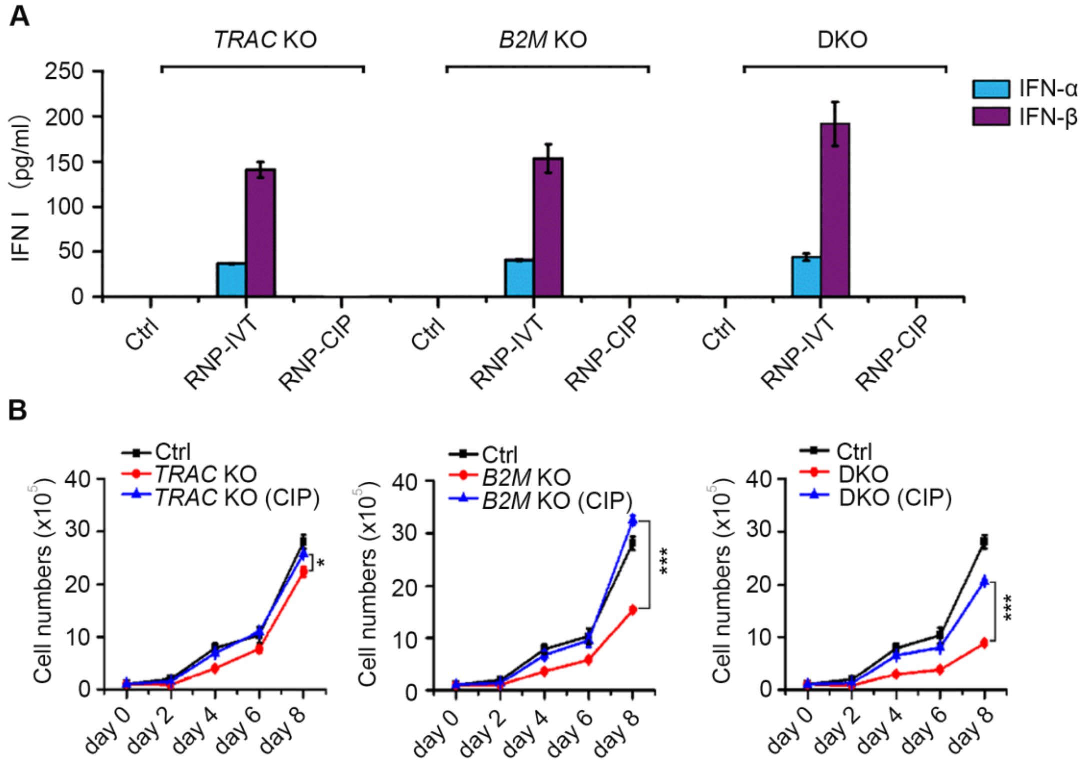

Supplemental Figure 8 | Removing 5' triphosphate of IVT sgRNA by CIP rescued detrimental effects in CAR-T cells. (A) The IFN I production of CAR-T cells after electroporation with indicated RNPs. Error bars represent SD for three technical replicates. **(B)** CIP treatment improved the proliferation of gene edited CAR-T cells (Mean ± SD, n = 3). 1×10^5 cells were electroporated with 6 μg indicated sgRNAs or RNPs. *$P < 0.05$, ** $P < 0.01$, *** $P < 0.001$, P values were calculated by employing unpaired one-way ANOVA test comparing with the values from indicated groups. DKO represents *TRAC-B2M* double knockout.

Figure S9

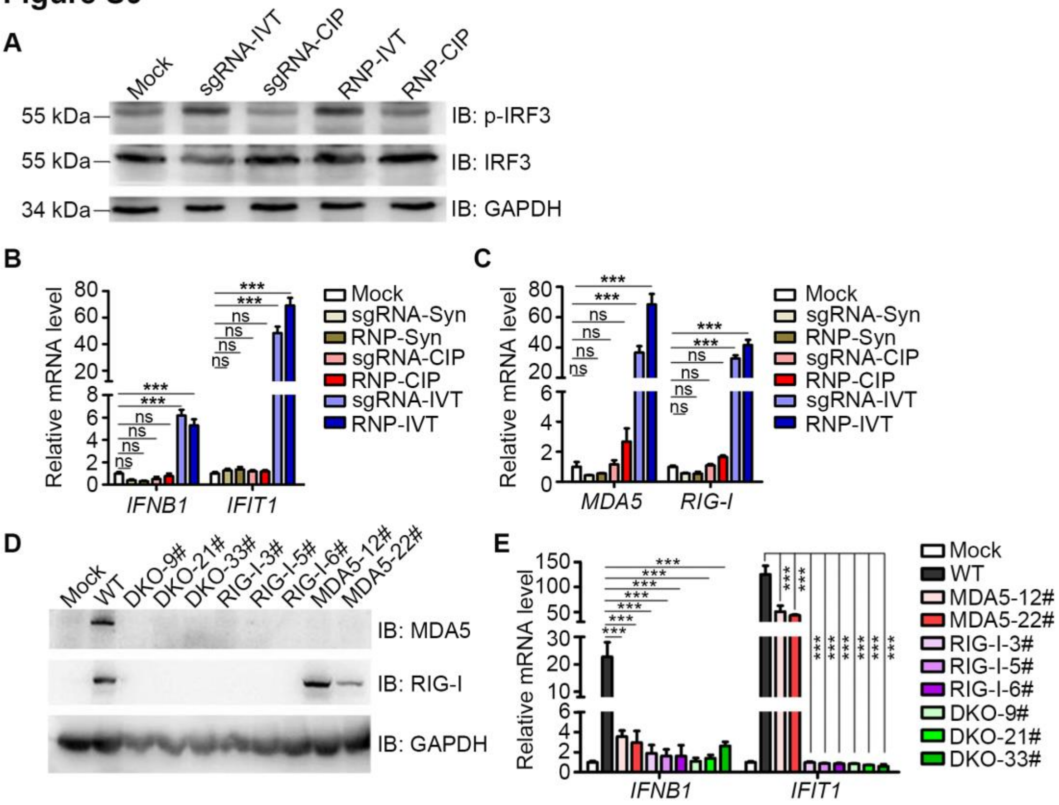

Supplemental Figure 9 | IVT sgRNA induced innate immunity response via MDA5 and RIG-I signaling pathway in Hela cells. (A) The phosphorylation level of IRF3 in Hela cells 24h after indicated sgRNA electroporation. **(B)** The mRNA expression levels of *IFNB1*and *IFIT1*in Hela cells 24h after indicated *AAVS1* sgRNA electroporation. **(C)** The mRNA expression levels of *MDA5* and *RIG-I* in Hela cells 24h after indicated *AAVS1* sgRNA electroporation. **(D)** The protein levels of RIG-I and MDA5 in wild type (WT), *MDA5* KO, *RIG-I* KO and DKO Hela cells 24h after *AAVS1* IVT sgRNA electroporation. **(E)** The mRNA expression level of *IFNB1*and *IFIT1* in wild type (WT), *MDA5* KO, *RIG-I* KO and DKO Hela cells 24h after *AAVS1* IVT sgRNA electroporation. sgRNA-Syn: Chemically synthetic 5'-OH *AAVS1* sgRNA; RNP-Syn: Chemically synthetic 5'-OH *AAVS1* sgRNA + Cas9 protein. DKO:

MDA5 and *RIG-I* double knockout. Error bars represent SD for three technical replicates. *P* values were calculated by employing unpaired one-way ANOVA test comparing the values from indicated group. ***$P < 0.001$. ns, no significant.

Fugure S10

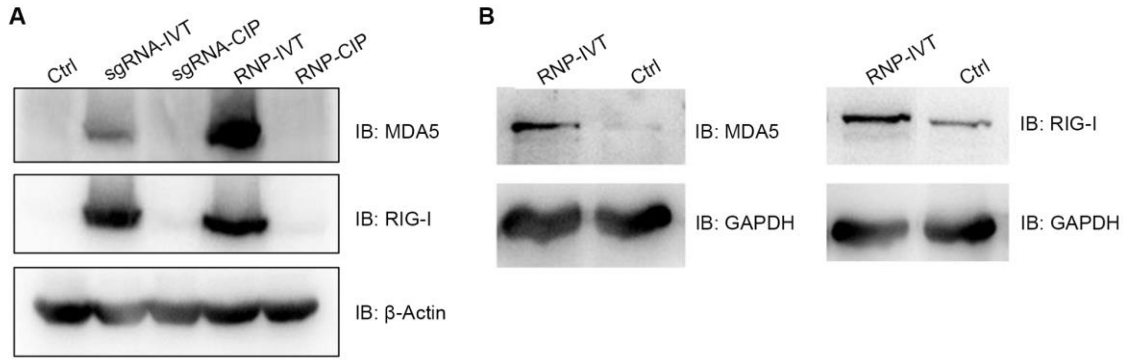

Supplemental Figure 10 | IVT sgRNA induced endogenous expression of MDA5 and RIG-I protein in Hela and CD3$^+$ T cells. The protein levels of MDA5 and RIG-I in Hela cells **(A)** and primary CD3$^+$ T cells **(B)** 24h after *AAVS1* IVT sgRNA electroporation were monitored by western blot with β-Actin or GAPDH as an internal control respectively.

Figure S11

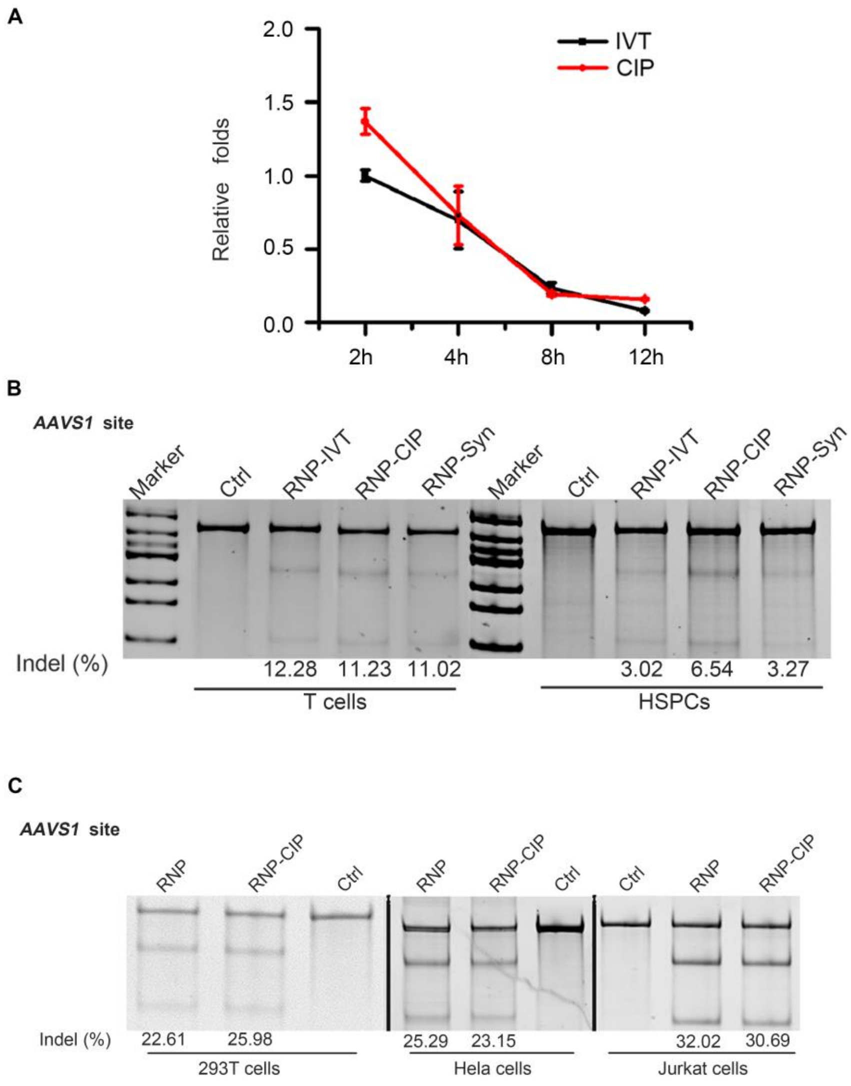

Supplemental Figure 11 | CIP treatment did not affect the stability of sgRNA and gene editing efficiency of CRISPR-Cas9 system. (A) The existence of *AAVS1* IVT sgRNAs, with or without CIP treatment, was measured by qPCR at different time points after transfection in 293T cell line. **(B)** The gene editing efficiencies at targeted sites by CIP treated or untreated RNPs were measured by surveyor assay in T and

HSPCs. **(C)** The gene editing efficiencies of *AAVS1* IVT sgRNAs, with or without CIP treatment, were detected by Surveyor assay in 293T, Hela and Jukat cell lines.

Figure S12

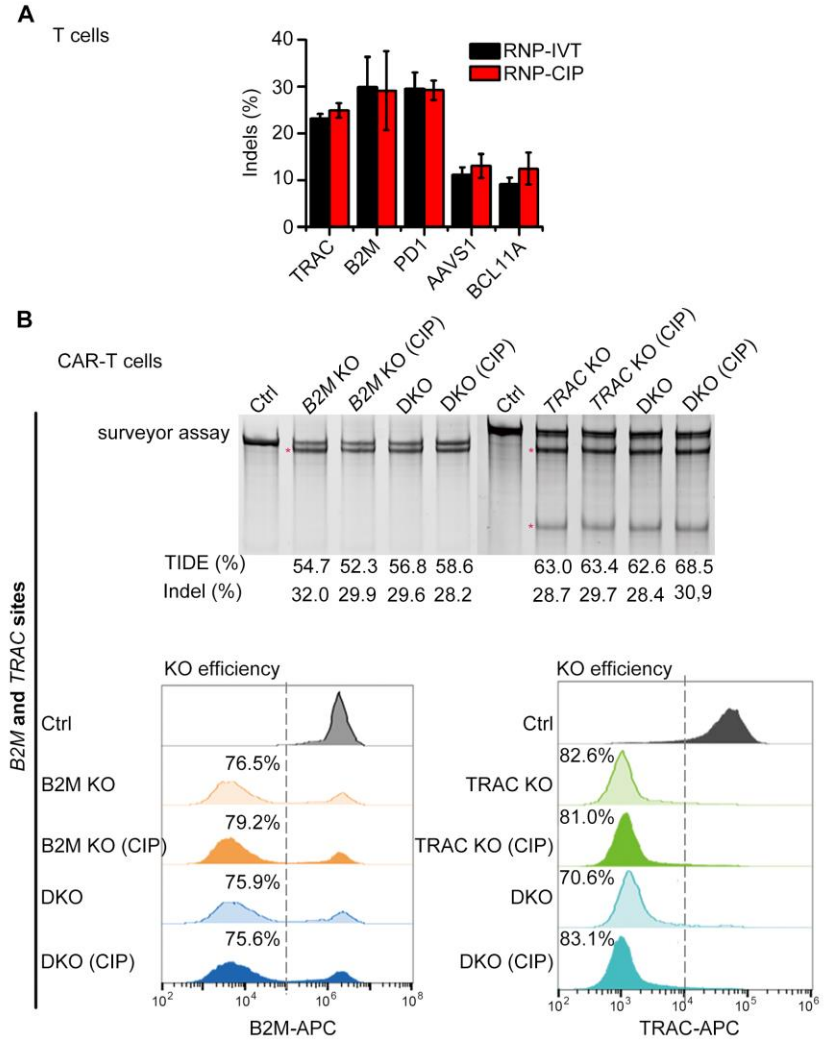

Supplemental Figure 12 | CIP treatment did not affect the gene editing efficiency of CRISPR-Cas9 system. (A) CIP treatment did not affect the gene editing efficiency of CRISPR-Cas9 system in $CD3^+$ T cells. Indel frequencies were measured by Surveyor assay and bars represent average indel frequencies ±SD, n= 3. **(B)** CIP treatment did not affect the gene editing efficiency of CRISPR-Cas9 system in CAR-T cells. Indel frequencies were measured by Surveyor assay and TIDE sequencing at DNA level and the protein levels of target genes were measured by FACS. DKO represents *TRAC* and *B2M* double knockout.

Figure S13

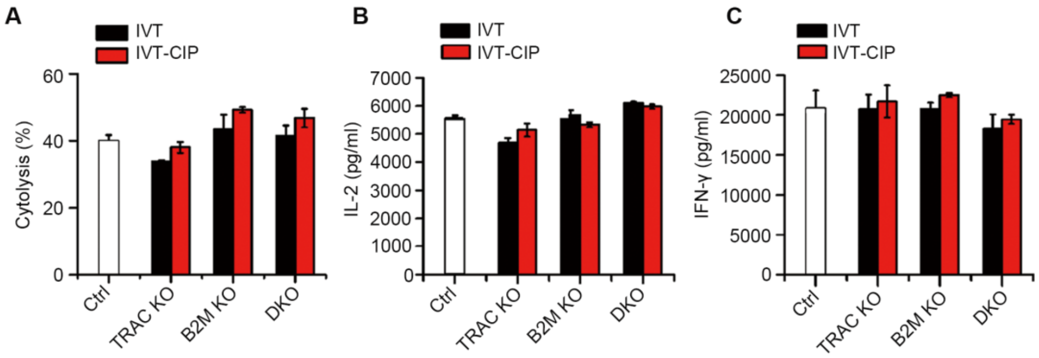

Supplemental Figure 13 | CIP treatment did not affect the functions of gene edited CAR-T cells.

(A, B, C) Luciferase based cytotoxicity **(A)** and cytokines release assay **(B, C)** evaluating the cell lytic activity of knockout CAR-T cells by CIP treated or untreated RNPs (Mean ± SD, n = 4). DKO represents TRAC-B2M double knockout.

Supplemental Table 1 | Sequences of sgRNA guide and DNA oligos used in this study

**Guide sgRNA sequence**	
B2M sgRNA	cgcgagcacagctaaggcca
TRAC sgRNA	acaaaactgtgctagacatg
PD1 sgRNA	cgactggccagggcgcctgt
BCL11A sgRNA	ctaacagttgcttttatcac
AAVS1 sgRNA	gacagaaaagccccatcctt
CCR5 sgRNA	ggcagcatagtgagcccaga
SOX2-1 sgRNA	gctgccgggttttgcatgaa
SOX2-2 sgRNA	gccgggttttgcatgaaagg
OCT4-1 sgRNA	ggtggtggcaatggtgtctg
OCT4-2 sgRNA	gacacaactggcgcccctcc
RIG-I sgRNA	cgtggcgagcggggaaagca
MDA5 sgRNA	atagcggaaattctcgtctg
***In vitro* transcription primers**	
Forward	taatacgactcactatagNNNNNNNNNNNNNNNNNNNNgtttaagagctatgctggaaac
Reverse	aaaagcaccgactcggtgcc
**Genotyping primers for surveyor assays**	
B2M-Forward primer	aatataagtggaggcgtcgc
B2M-Reverse primers	cgcgttcacaaacctcagc
TRAC-Forward primer	atatccagaaccctgaccctgc
TRAC-Reverse primer	ggcaggcaggaactcagttg
PD1-Forward primer	cctgtctctgtctctctctcc
PD1-Reverse primer	gccagggactgagagtgaaag
BCL11A-Forward primer	aaagcgatacagggctgg
BCL11A-Reverse primer	ggtgcctatatgtgatggatgg
AAVS1-Forward primer	cttacctctctagtctgtgctagc
AAVS1-Reverse primer	ggatcctctctggctccatcg
**Primers for qPCR**	
GAPDH-Forward primer	atgacatcaagaaggtggtg
GAPDH-Reverse primer	cataccaggaaatgagcttg
IFNB1-Forward primer	aggacaggatgaactttgac
IFNB1-Reverse primer	tgatagacattagccaggag
IFIT1-Forward primer	ccttgggttcgtctacaaattg
IFIT1-Reverse primer	gtgaaagtggctgatatctgg

RIG-I-Forward primer	ggcattgacattgcacagtg
RIG-I -Reverse primer	gtcagaaggaagcacttgct
MDA5-Forward primer	ggcaccatgggaagtgatt
MDA5-Reverse primer	tttggtaaggcctgagctg

REFERENCE

Brinkman, E.K., Chen, T., Amendola, M., and van Steensel, B. (2014). Easy quantitative assessment of genome editing by sequence trace decomposition. Nucleic Acids Res 42, e168-e168.

Guschin, D.Y., Waite, A.J., Katibah, G.E., Miller, J.C., Holmes, M.C., and Rebar, E.J. (2010). A Rapid and General Assay for Monitoring Endogenous Gene Modification. In Engineered Zinc Finger Proteins: Methods and Protocols, J.P. Mackay, and D.J. Segal, eds. (Totowa, NJ, Humana Press), pp. 247-256.

Liu, X., Zhang, Y., Cheng, C., Cheng, A.W., Zhang, X., Li, N., Xia, C., Wei, X., Liu, X., and Wang, H. (2016). CRISPR-Cas9-mediated multiplex gene editing in CAR-T cells. Cell Res 27, 154.

Moon, E.K., Wang, L.-C., Dolfi, D.V., Wilson, C.B., Ranganathan, R., Sun, J., Kapoor, V., Scholler, J., Puré, E., Milone, M.C., *et al.* (2014). Multifactorial T cell Hypofunction that is Reversible Can Limit the Efficacy of Chimeric Antibody Receptor-transduced Human T cells in Solid Tumors. Clinical cancer research : an official journal of the American Association for Cancer Research 20, 4262-4273.
